# Gold- and Silver Nanoparticles Affect the Growth Characteristics of Human Embryonic Neural Precursor Cells

**DOI:** 10.1371/journal.pone.0058211

**Published:** 2013-03-11

**Authors:** Erika Söderstjerna, Fredrik Johansson, Birgitta Klefbohm, Ulrica Englund Johansson

**Affiliations:** 1 Institute of Clinical Sciences, Department of Ophthalmology, Lund University, Lund, Sweden; 2 Department of Biology, Section of Functional Zoology, Lund University, Lund, Sweden; University of California, Merced, United States of America

## Abstract

Rapid development of nanotechnologies and their applications in clinical research have raised concerns about the adverse effects of nanoparticles (NPs) on human health and environment. NPs can be directly taken up by organs exposed, but also translocated to secondary organs, such as the central nervous system (CNS) after systemic- or subcutaneous administration, or via the olfactory system. The CNS is particularly vulnerable during development and recent reports describe transport of NPs across the placenta and even into brain tissue using *in vitro* and *in vivo* experimental systems. Here, we investigated whether well-characterized commercial 20 and 80 nm Au- and AgNPs have an effect on human embryonic neural precursor cell (HNPC) growth. After two weeks of NP exposure, uptake of NPs, morphological features and the amount of viable and dead cells, proliferative cells (Ki67 immunostaining) and apoptotic cells (TUNEL assay), respectively, were studied. We demonstrate uptake of both 20 and 80 nm Au- and AgNPs respectively, by HNPCs during proliferation. A significant effect on the sphere size- and morphology was found for all cultures exposed to Au- and AgNPs. AgNPs of both sizes caused a significant increase in numbers of proliferating and apoptotic HNPCs. In contrast, only the highest dose of 20 nm AuNPs significantly affected proliferation, whereas no effect was seen on apoptotic cell death. Our data demonstrates that both Au- and AgNPs interfere with the growth profile of HNPCs, indicating the need of further detailed studies on the adverse effects of NPs on the developing CNS.

## Introduction

Nanotechnology and nanobioscience are major research areas that are rapidly expanding. Recent advances in these areas have stimulated new applications within biomedicine where nanomaterial can be used to achieve a more effective and safe drug delivery approach. A nanomaterial is by definition an object with at least one dimension in the range of 1–100 nm, which includes nanogels, nanofibers, nanotubes and nanoparticles (NPs, *e.g.* rods, cubes, and spheres) [1], [2]. NPs can be manufactured from a wide range of materials *e.g.* polymers, metals, carbon, silica, and materials of biological origin such as lipids or lactic acid. Engineered NPs are attractive for medical purpose due to their translocational properties in tissue and the fact that their surface to volume ratio is larger than for microsized particles and hence the ability to adsorb and carry other compounds [3]. NPs can also serve as probes appropriate for different medical purposes, *e.g.* imaging thermotherapy. Furthermore, nanomaterial is increasingly used in a variety of commercial products including clothing, cosmetics, electronics, and food [1], [4], and therefore the risk of unintentional exposure becomes obvious.

The small size of NPs make them more reactive due to the larger surface area per volume and therefore such particles may enhance the desired effects, as mentioned above, but also new unwanted toxic effects may be introduced [5]. Especially, two metal NPs, Au- and AgNPs, have been intensively studied, AuNPs due to their good intrinsic properties such as high chemical stability, well-controlled size and surface functionalization, and AgNPs, due to their antibacterial effect, often applied in wound disinfection, in coatings of medical devices and prosthesis, and commercially in textiles, cosmetics and household goods [6]. Au has been widely described as highly biocompatible and Au-based NPs have been extensively investigated and also clinically used in drug and gene delivery applications [7].

However, it has become evident that the field of nanotoxicology is lagging the study of biomedical applications of different NPs. To avoid repeating historical mistakes such as asbestosis or silicosis, it is crucial to unravel the possible toxic effects of NPs before they are spread into the ecosystems and become a common health issue in our society [8]. A very recent report shows that commercially manufactured polystyrene NPs can be transported through an aquatic food chain from algae, through zooplankton to fish, and affect lipid metabolism and behavior of the top consumer [9]. One should bear in mind that the accumulation of other heavy metals, such as Hg in ecosystems, has lead to a common acceptance to decrease intake of top consumer fish types [10]. This is especially true for pregnant women where excessive intake of heavy metal-containing fish can cause detrimental damage to the developing fetus. Today, the knowledge on human exposure and possible toxicity of engineered NP-based products are very limited. Therefore, investigations on the biokinetics of different NPs in an organism is currently given much attention since there is an urgent need for information on the absorption, distribution, metabolism and excretion (ADME) of NPs and validated detection methods of engineered nanoparticles.

The number of reports describing unwanted non-target effects of various NPs is increasing. In experimental studies it has been described that NPs can cause adverse effects not only to primary organs directly exposed, but also to secondary organs, such as the cardiovascular system and the central nervous system (CNS) [11]. Neuronal systems are especially vulnerable, both during development but also in adulthood, to metal intoxication that have been associated with the development of the major neurodegenerative diseases, such as Alzheimer’s- and Parkinson’s diseases [4], [12].

The adult CNS can be reached both by AuNPs and AgNPs through different routes in the body and reports on their cytotoxic effects are increasing [13]. Nanosized Au has recently been found to have damaging effect on epithelial cells and a human carcinoma cell line [14], [15], [16], [17]. AuNPs have also been reported to pass the blood-retinal-barrier (BRB) after intravenous injection in mice [7], [18]. Moreover, AgNPs have been reported to pass the blood-brain-barrier (BBB), *i.e.* manganese oxide- and AgNPs, respectively, reach the CNS after inhalation and subsequently uptake in the respiratory tract [19], [20]. Moreover, after both oral intake and intravenous administration, Au- and AgNPs have been reported to reach several secondary organs, including the CNS [21], [22]. Observed pathological effect in brain tissue to NP exposure has mainly been oxidative stress and inflammation [19], [23], [24], [25], [26].

If there are concerns about the toxic effect of NPs to the adult CNS, the concerns should be even greater with regard to possible risk of translocation of NPs across the placenta and effects on embryogenesis. Little is currently known about whether NPs can cross the human placental barrier or interfere with placental function, but suitable transport models have been developed which can be used to clarify the mechanisms of cellular interaction and transport across the placenta. A few investigations provide evidence for transport of commonly used NPs, such as silica, TiO_2_- and cadmium-NPs, which hence warrants further investigation into the potential of various NPs to possibly affect embryogenesis [27], [28], [29], [30], [31], [32], [33].

Even at low concentrations toxic NPs accumulation inside the cells during development can have a detrimental impact on the fetus development and thereby the viability of the post-natal fetus. For the developing CNS, cell division, cell differentiation and cell migration follow a highly coordinated scheme where DNA needs to be replicated, proteins distributed to cells in a controlled fashion, cells need to divide at the right point in time, and so forth. All these processes are extremely prone to the slightest changes occurring in the intra- or extracellular environment.

Up-to-date, a sparse number of *in vitro* studies have been published using neural-like cell lines for testing the effect of NPs. Studies including PC-12 cells (a rat cell line with a neuronal-like phenotype) have shown changes in gene expression and interference with signal transduction pathways after Cu- and AgNPs exposure [34], [35]. Two different reports using primary neural cultures from mice demonstrated interference with electrical activities after treatment with Ag-, TiO_2_-, carbon black-, or hematite- derived NPs [36], [37], [38]. Lately, oxidative stress responses including interference with calcium-based signaling processes were further reported after AgNP exposure, using mixed primary neural cultures [39]. Furthermore, in a multiparametric study AuNP exposure affected cellular proliferation and differentiation in an immortalized neural cell line (C17.2), primary HUVECs and PC12 cells [17].

Here, for the first time, we analyze the effect of low concentration Au- and AgNPs on the growth characteristics of a human CNS-derived neural precursor cell line. The human neural cells are cultured as neurospheres (aggregates of free-floating cells), thus serving as a good model of a three-dimensional neural system under development. The cell line is established from cells isolated from seven week old fore-brain and can be expanded into high numbers in the presence of epidermal growth factor (EGF), human basic fibroblast growth factor (hbFGF) and human leukemia inhibitor growth factor [40], [41]. We studied the eventual cytotoxic effects on these cells after two weeks exposure of 20 and 80 nm Au- and AgNPs at low concentrations (ranging from 0.00022–0.22 µg/ml). Low concentrations of the NPs were used to mimic the low fraction of NPs reported to reach secondary organs (0.1–3%) [42], [43]. The following end-points were used: 1) uptake of NPs was studied using transmission electron microscopy (TEM), 2) gross- and detailed morphological studies were performed using routine Htx-Eosin staining as well as scanning electron microscopy (SEM), 3) ratio of viable vs. dead cells was calculated by counting cytochemical stained cells 4) numbers of proliferative cells (Ki67 immunostaining), and dying apoptotic cells (TUNEL assay) were quantified.

For the first time, we describe uptake of both 20 and 80 nm Au- and AgNPs, by HNPCs under proliferation conditions. Our data demonstrate that both Au- and AgNPs interfere with the growth profile of human neural precursor cells, indicating the importance of further detailed studies on the adverse effects of NPs on the developing CNS.

## Results and Discussion

### Translocation of NPs Over the Placenta and to Secondary Organs

There are several primary routes for NPs to enter the body; inhalation, via the skin or orally, as well via the eye. From these primary routes many types of NPs has been described to reach also secondary organs (passing through important barriers *e.g.* BBB), such as the cardiovascular system and the CNS [13], [19], [20], [21], [22]. In addition, over the last five years reports have emerged describing a detrimental effect on embryogenesis and fetus vitality after NP exposure of pregnant rodents, indication NP translocation over the placenta [31], [32]. NPs were found localized in the developing CNS, suggesting a risk of interference with the complex events responsible for normal CNS development. In agreement, several studies demonstrate that NPs exposure cause oxidative stress and inflammatory response using *in vitro* models of neural systems [23], [24], [25]. Such pathological conditions are well known to be associated with the progression of the most common neurodegenerative diseases, including Alzheimer’s- and Parkinson’s diseases [4], [12].

Investigations using models of neural system-resembling cell assays begin to gather evidence on the great impact even very low concentrations of NPs [43]. The limited numbers of reports elucidating the key mechanisms for NP-related CNS cytotoxicity often rely on the neuronal-like rat PC-12 cell line or other immortalized neural cell lines, such as C17.2 [17]. Since immortalized cell lines have mutated cellular functions, such as disturbed cell proliferation and cell death mechanisms, the use of these types of cells in toxicological studies are questionable. Several others have shown that NPs may or may not affect the cell proliferation and cell death characteristics of tumour cells, however, these reports cannot totally be related to cell growth characteristics of normal cells. Today, there are NP-based chemotherapeutic agents on the market even though the impact NPs have on normal cells is not fully understood [44]. Here, for the first time, we use a human neural precursor cell- based assay to gain knowledge on how normal cells are affected by Au- or AgNPs. The cell line can be regarded as model of a developing brain, since the cells originate from forebrain tissue, obtained from one 7-week (post-conception) human embryo and are grown as a so-called neurosphere culture. The cell line can be cultured and expanded for at least one year *in vitro* with maintained multipotentcy [40], [45]. HNPCs grown as neurospheres have been used in developmental cytotoxicity (DCT) studies for environmental chemicals. The use of HNPC lines as a model for mimicking basic processes of brain development; such as cell proliferation, differentiation, migration and apoptosis, has proven to be a useful tool [46].

We here use two different types of NPs, Au since it is believed to be inert and suggested for different therapeutical applications, and Ag since it is known to have toxic effects and is widely used in consumer products. The diameter of the NPs as measured in transmission electron microscope (TEM) was 20 and 80 nm. The NPs were used in low concentrations since it has been reported that low levels of NPs were found in secondary organs after systemic administration using different study designs [42], [43]. We also included silver nitrate (AgNO_3_), as a positive control for cytotoxicity, since the release of Ag^+^ ions is known to be very toxic. An overall goal with the present and on-going studies using the current HNPC-based assay is to establish a battery of appropriate end-points to be utilized in further investigations on cellular effects of other NPs. This cell line can also be used for testing cell effects both during proliferation and differentiation conditions.

Light microscopy was used to study both gross and detailed morphology after Htx-eosin staining, and scanning electron microscopy (SEM) was used to study detailed surface morphology of the neurospheres.

### Cellular Uptake of Au- and AgNPs by HNPCs

**Figure 1:**
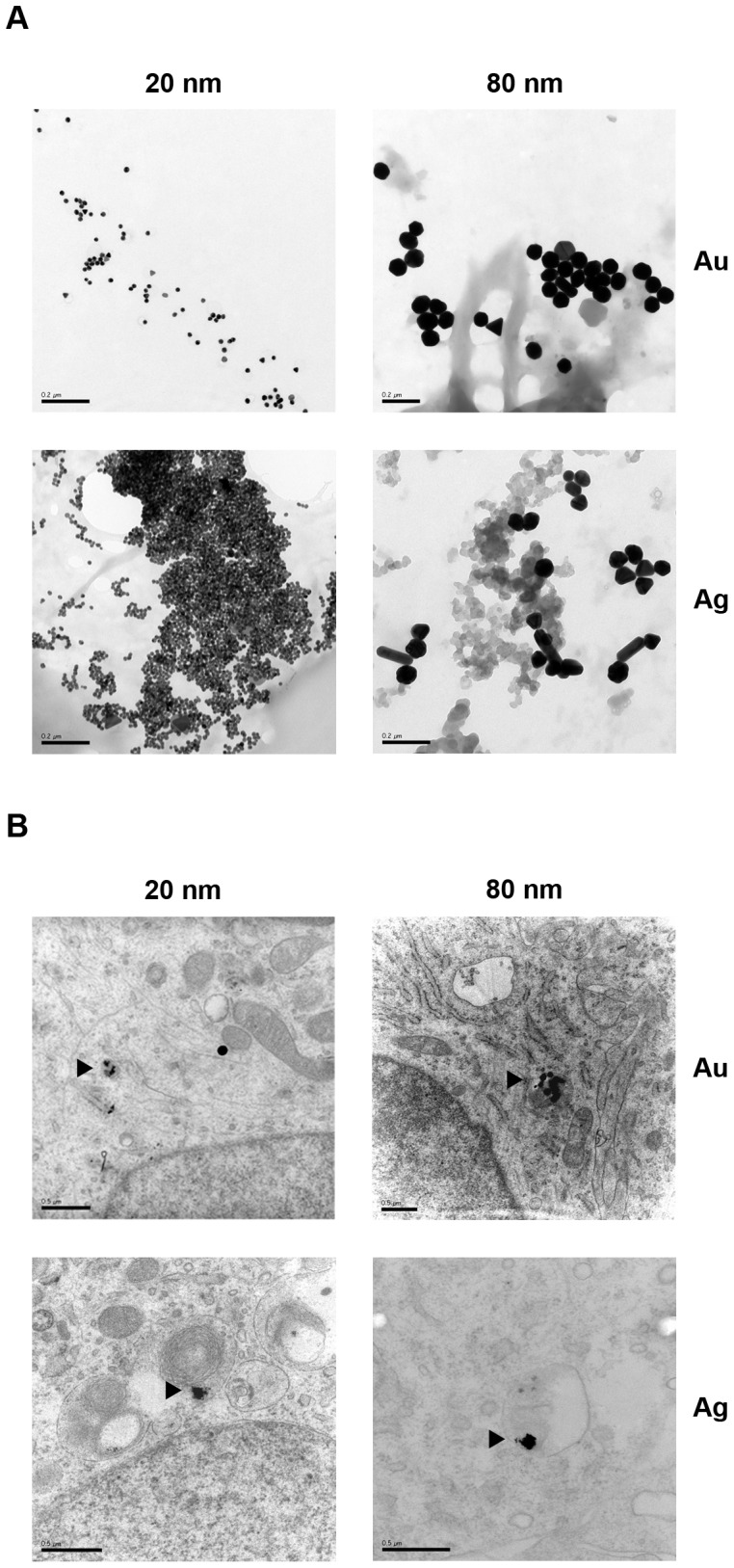
TEM evaluation of the uptake of Au- and AgNPs of HNPCs. A. Gold and silver NPs (20 and 80 nm) imaged by TEM. Scale bars equal 0.2 µm. **B.** NPs were added to the medium 48 h after seeding and spheres were prepared for transmission electron microscopy two weeks later. All four different types of NPs were taken up by HNPCs. Arrowheads show the NPs that have been taken up by HNPCs. Scale bars equal 0.5 µm.

First we investigated, by using TEM, whether the current used HNPCs had the ability to internalize Au- and AgNPs. Since AuNPs and AgNPs have high atomic numbers it is possible to distinguish them from cellular structures using TEM [47], [48]. We demonstrate that 20 and 80 nm Au- and AgNPs can be taken up by human neural cells under expansion conditions, a crucial finding for all our further investigations on NP exposure effect on HNPCs (Fig. 1). In agreement, recent studies showed that mesenchymal stem cells (MSC), neural progenitor cells, and HeLa cells can take up 20 nm coated- and uncoated AuNPs in the same size range as used here [17], [49], [50].

Au- and AgNPs of both sizes were only found in various cellular regions, including the cytosol, except the nucleus, judged from scanning through the entire spheres including detailed high magnification analysis of at least 50 cells/experimental group. Here, the NPs were found as compact aggregates in the cytoplasm of the cells, as has been reported by others [17], [50], [51], [52]. This finding is in line with many reports on cellular localisation of ingested NPs in the cytoplasm [17], [50]. In contrast, Hackenberg *et al* showed uptake of AgNPs (<50 nm) also in the nucleus of MSCs [51].

Dziendtzowska *et al* [21] demonstrated that AgNPs are able translocate from the blood to the main organs and the concentration of silver in tissues was significantly higher in rats intravenously injected with 20 nm AgNPs compared with 200 nm AgNPs. The silver concentration in the kidneys and brain increased during their experiment and reached the highest concentration after 28 days [21]. Heavy metals are known to be hazardous and studies both *in vitro* and *in vivo* implicate that Au- and AgNPs are able to pass both the intact BBB [53], [54] and the BRB [18]. Typical pathological signs such as inflammation and oxidative stress have been described in the rodent brain after manganese oxide and silver exposure [19], [26].

### NP Exposure Affects Sphere Size and Sphere Aggregation Properties

**Figure 2:**
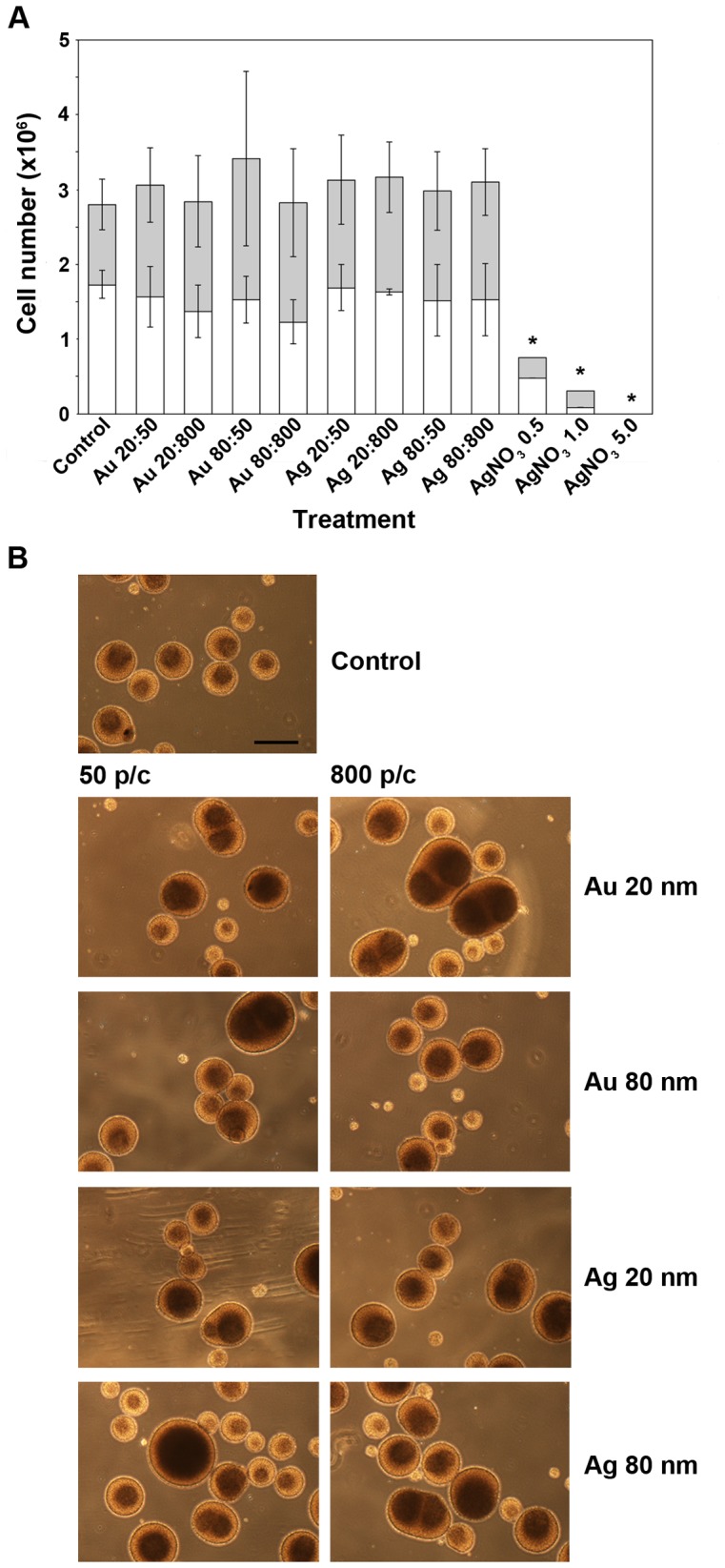
Effects on cell growth after Au- or AgNPs of HNPCs. **A.** Au- or AgNPs did not significantly affect the cell growth, presented as total numbers of live and dead cells, of HNPCs. The results are presented as mean values (n = 2−3 samples from three independent experiments). Error bars represent ± SD. White bars, live cells; grey bars, dead cells; Au 20∶50, gold 20 nm and 50 particles/cell; Au 20∶800, gold 20 nm and 800 particles/cell; Au 80∶50, gold 80 nm and 50 particles/cell; Au 80∶800, gold 80 nm and 800 particles/cell; Ag 20∶50, silver 20 nm and 50 particles/cell; Ag 20∶800, silver 20 nm and 800 particles/cell; Ag 80∶50, silver 80 nm and 50 particles/cell; Ag 80∶800, silver 80 nm and 800 particles/cell; AgNO_3_ 0.5, silver nitrate 0.5 mg/ml; AgNO_3_ 1.0, silver nitrate 1.0 mg/ml; AgNO_3_ 5.0, silver nitrate 5.0 mg/ml. *p<0.05 compared to control. **B.** Both Au- and AgNPs affect the morphology of HNPCs when grown as neurospheres. AgNO_3_ was toxic to HNPCs. NPs or AgNO_3_ were added 48 h after seeding. The shown data are representative of three independent experiments. 50 p/c, 50 particles/cell; 800 p/c, 800 particles/cell; Scale bar equals 400 µm.

After two weeks of exposure to NPs or AgNO_3_, images of the HNPC neurospheres were acquired for size measurements. The spheres were thereafter dissociated and total cell numbers were determined. AuNPs or AgNPs did not significantly affect the total number of living and dead cells, respectively, compared to control (Fig. 2A). The fraction of dead NP-exposed cells, identified by Trypan Blue (TB) staining, ranged from 46–56% of the total cell number as compared to control where this fraction was 46%. An increase of up to 22% of dead cells as compared to control was shown after NP-exposure (n = 2−3). However, the rather high number of dead cells also found in the control may result from the mechanical dissociation used to dissociate the cells before counting. TB staining is a commonly used method for excluding viable cell from dead cells, since TB is unable to pass the intact cell membrane of viable cells. In the current study, only low concentrations of the NPs were used and our results show no effect on cell viability judged from the cell counting, while others have used higher NP concentrations and have reported that cell viability decrease with increased concentration or incubation time of AgNPs [55], [56]. For example, Soenen *et al* show that cell viability decrease after exposure to large AuNPs, *i.e.* 200 and 500 nm, but not with smaller AuNPs [17]. In parallel, AuNPs have not been reported to alter cellular functions after two weeks of exposure [51], while AgNPs on the other hand have been shown to decrease the cell viability of MSC [49].

Observations from three independent experiments indicated that exposure to all NPs and all concentrations used resulted in an increased sphere aggregation and darker spheres cores, by using phase contrast microscopic analysis, compared to control. After NP-exposure, the majority of the spheres were larger; approximately 450 µm in diameter although with a larger variation as compared to 335 µm of the control spheres (Fig. 2B). Some of the spheres exposed to NPs were up to twice the size, 700 µm, of control spheres. The smaller spheres found after NP-exposure were in the size-range of 200–270 µm. Although, the increase in sphere aggregation and overall larger spheres were more profound after AgNP-exposure, while AuNP-exposure resulted in darker sphere cores, and some of the spheres were twice as large compared to control spheres. For all four NPs, the concentration seems to affect the outcome in a dose-response fashion, 800 particles/cell (p/c) had more effect than 50 p/c on sphere aggregation and sphere size variation. AgNO_3_ was used as a positive control since it is known to have a severe cytotoxic effect (although the responsible mechanism for this effect is not fully understood), which was here confirmed (Fig 2A). The NPs used in this study are dissolved in water. In our recent studies the NPs were centrifuged and discarded, with only the supernatant remaining (unpublished results). The supernatant was added to microglial cells and a cytotoxic test (MTT) was performed. The supernatant alone did not induce toxicity. Therefore, we expect no toxicity associated with the vehicle. As mentioned above, NPs are known to often form aggregates and they do so when they come in contact with biological fluid. The material, size and surface may alter the biological nature of the proteins in the corona, and subsequently the biological impact [57], [58]. This change of protein action in the corona could be one reason to the differences in size and the different ability of the NP-exposed neurospheres to form aggregates consisting of several spheres closely attached to each other. Together, our findings that Au- and AgNP affect HNPC normal growth are very important with regard to the studies showing that NPs can indeed cross the placenta and inhalation of cadmium oxide NP during pregnancy affects the reproductive fecundity, hence alters fetal and postnatal growth of the developing offspring [33], [59]. Thus, our results on changes in human neurosphere size and capacity of neurosphere-neurosphere aggregations suggest that Au- and AgNP exposure could affect the basic growth characteristics of a three-dimensional developing human CNS.

### Au- and AgNPs Affect both Cell Proliferation and Apoptosis of HNPCs

Since no significant difference were found when counting the total numbers of HNPCs after two weeks of NP-exposure, despite a clear overall morphological difference compared to control cultures was observed when studying the neurosphere sizes and composition of small and large spheres, we decided to study expression pattern of markers for cell proliferation and apoptosis, respectively.

**Figure 3:**
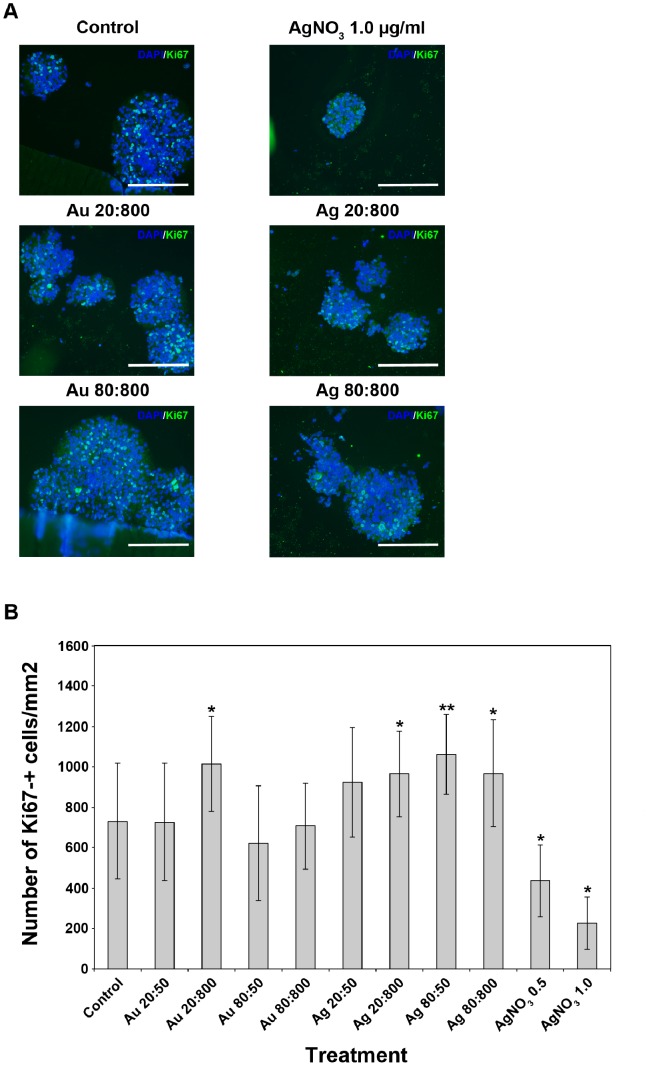
NP exposure increases numbers of HNPC proliferating Ki67-+ cells. A. AgNP-exposure for two weeks increased the cell proliferation in HNPCs, seen as increased numbers of Ki67+ cells (green). Representative images of 6 spheres per section from two independent experiments. Blue staining, DAPI. Au 20∶800, gold 20 nm and 800 particles/cell; Au 80∶800, gold 80 nm and 800 particles/cell; Ag 20∶800, silver 20 nm and 800 particles/cell; Ag 80∶800, silver 80 nm and 800 particles/cell; AgNO_3_ 1.0, silver nitrate 1.0 mg/ml. Scale bars equal 214 µm. **B.** Only AgNPs significantly induced cell proliferation in HNPCs. Evaluation of the data presented under A, showing the number of Ki67-+ cells/mm^2^ of the HNPC neurospheres. Au 20∶50, gold 20 nm and 50 particles/cell; Au 20∶800, gold 20 nm and 800 particles/cell; Au 80∶50, gold 80 nm and 50 particles/cell; Au 80∶800, gold 80 nm and 800 particles/cell; Ag 20∶50, silver 20 nm and 50 particles/cell; Ag 20∶800, silver 20 nm and 800 particles/cell; Ag 80∶50, silver 80 nm and 50 particles/cell; Ag 80∶800, silver 80 nm and 800 particles/cell; AgNO_3_ 0.5, silver nitrate 0.5 mg/ml; AgNO_3_ 1.0, silver nitrate 1.0 mg/ml. *p<0.05 compared to control, **p<0.01 compared to control.

In order to investigate if the cells had a disturbed cell proliferation, we estimated the relative numbers of Ki67-expressing cells in NP-exposed and control cultures, respectively (Figs. 3A, B). Changes in cell proliferation were examined by using immuno-labelling with a Ki67 antibody, a protein strictly associated with cell proliferation that can be exclusively detected in the nucleus during interphase [60]. For AuNP-exposed HNPC a significant increase in cell proliferation was only found after exposure to 20 nm AuNP using the highest concentration (Fig. 3B). However, a significant increase in cell proliferation was found after AgNP-exposure using 20 and 80 nm (both concentrations) as compared to control (Fig. 3B). As expected, the cell proliferation markedly decreased after AgNO_3_ exposure (Figs. 3A, B). Only few cells and no spheres were remaining after exposure to AgNO_3_ 5.0 µg/ml, hence no data or image are presented for this experimental group in any of the analysis presented hereafter. These findings can be directly correlated to the darker sphere cores and larger spheres present after NP exposure when imaging with light microscopy (Fig. 3B). The altered cell proliferation shown after NP exposure could be due to various stress factors. It has been shown that AuNPs can induce oxidative stress in neural progenitor cells [17] and AgNPs induce oxidative stress in skin fibroblasts [61].

**Figure 4:**
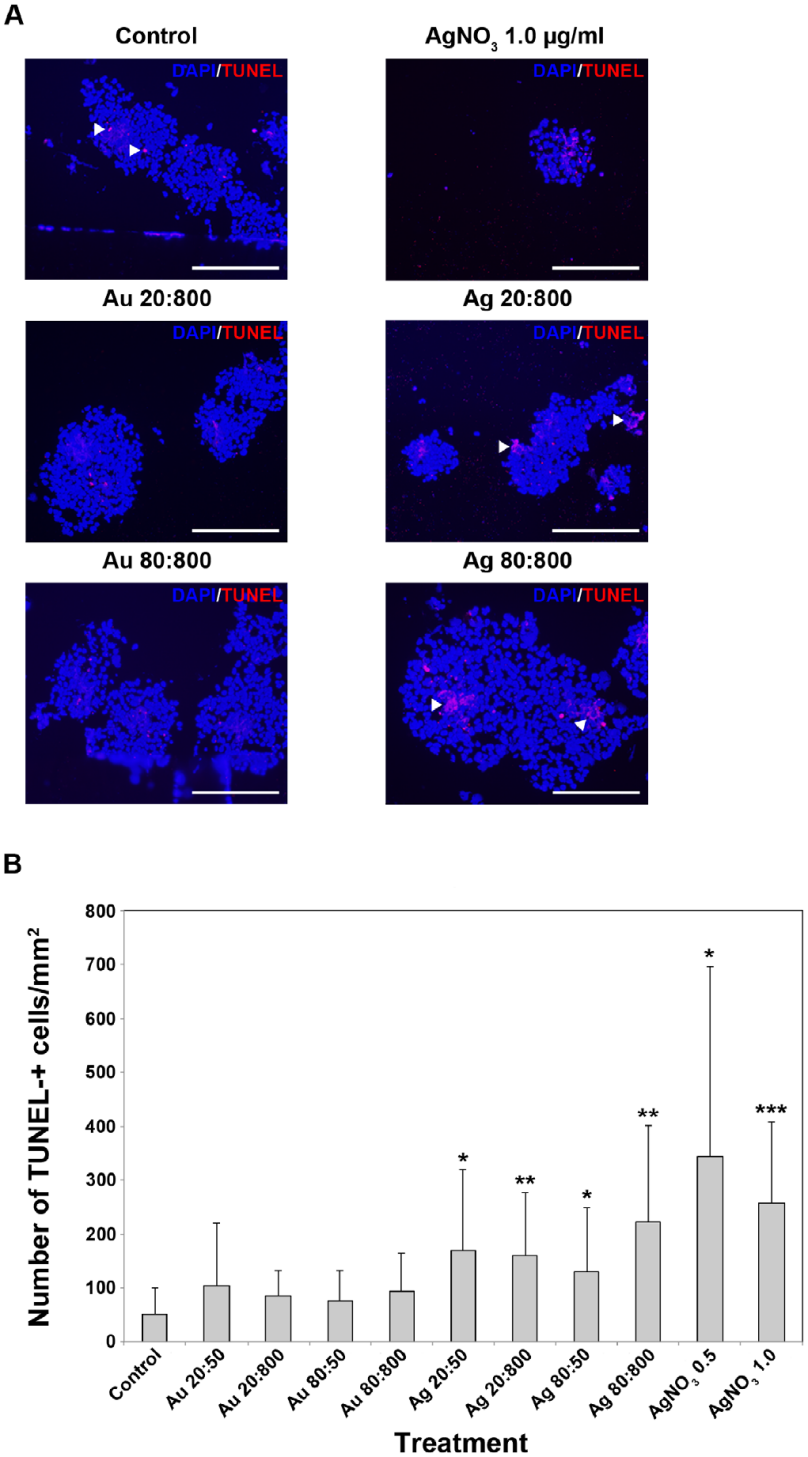
AgNP exposure increased numbers of apoptotic TUNEL+ HNPC. A. AgNP-exposure for two weeks increased the cell death in HNPCs, seen as increased number of TUNEL-+ cells. Both Au- and AgNPs induced cell death of HNPCs indicated by the increased number of TUNEL-+ cells and -aggregates. The presented data are representative images of 6 spheres per section from two independent experiments. Blue staining, DAPI; red staining, TUNEL; Au 20∶800, gold 20 nm and 800 particles/cell; Au 80∶800, gold 80 nm and 800 particles/cell; Ag 20∶800, silver 20 nm and 800 particles/cell; Ag 80∶800, silver 80 nm and 800 particles/cell; AgNO_3_ 1.0, silver nitrate 1.0 mg/ml. Scale bar equal 214 µm. **B.** Only AgNPs significantly induced cell death in HNPCs. Evaluation of the data presented under A, showing the number of TUNEL-+ cells/mm_2_ of the HNPC neurospheres. Au 20∶50, gold 20 nm and 50 particles/cell; Au 20∶800, gold 20 nm and 800 particles/cell; Au 80∶50, gold 80 nm and 50 particles/cell; Au 80∶800, gold 80 nm and 800 particles/cell; Ag 20∶50, silver 20 nm and 50 particles/cell; Ag 20∶800, silver 20 nm and 800 particles/cell; Ag 80∶50, silver 80 nm and 50 particles/cell; Ag 80∶800, silver 80 nm and 800 particles/cell; AgNO_3_ 0.5, silver nitrate 0.5 mg/ml; AgNO_3_ 1.0, silver nitrate 1.0 mg/ml. *p<0.05 compared to control, **p<0.01 compared to control, ***p<0.001 compared to control.

Direct cytotoxic effects of Au- or AgNPs on HNPCs, was investigated using a fluorescein-conjugated dUTP TUNEL-staining to detect cells undergoing apoptosis. TUNEL-staining is commonly used to detect DNA fragmentation, a hallmark for cells undergoing apoptotic cell death [62]. The number of TUNEL+ cells increased after exposure to either Au- or AgNPs, compared to control (Fig. 4A). However, the increase of TUNEL+ cells was only significant after exposure to AgNP (Fig. 4B). The increase of TUNEL+ cells seems to be correlated to increased concentration of the NPs. In approximately 30–50% of the large spheres dense aggregates of TUNEL+ cells were observed. The aggregates varied in size, from small aggregates containing only few cells to large aggregates containing up to hundreds of cells (Fig. 4B). These aggregates were found in both Au- or AgNP-exposed neurospheres. A DNA leakage from the nucleus into the cytoplasm during apoptosis, and further into the extracellular space within the spheres could explain the aggregate-like TUNEL-staining pattern [63], [64]. AgNO_3_-toxicity was confirmed by the significant and very large increase of TUNEL+ cells (Figs. 4A, B). Small spheres exposed to NPs resembled control spheres. This may suggest that smaller aggregates of HNPCs are less vulnerable for Au- and AgNP exposure, but this needs to be further explored.

Our results are in line with several recent reports using different assays describing interference with DNA regulation after NP exposure. In mouse embryonic stem cells, AgNPs induce apoptosis shown by increased Annexin V expression, up-regulation of p53, and DNA damage [56]. By using a lactate dehydrogenase (LDH) assay AgNPs was shown to induce cell death in a size and dose dependent manner in a primary mixed neural cell culture, while AuNPs had no affect [39]. The activation of the transcription factor NFκB, a critical immediate early response gene to injury, can another way to study apoptosis, and large AuNPs have been found to induce activation of the NFκB pathway due to the oxidative stress, while small AuNPs have no effect [17]. In summary, it is clear that AgNPs have the ability to increase apoptosis in different stem cell-based assays, again indicating the risk for a developing organism to be negatively affected by NP exposure.

### Gross- and Detail Morphological Studies of Human Spheres

The biology of NSCs, cultured as neurospheres *in vitro*, is still not fully understood. The NSCs and neurospheres are both functional and morphological heterogeneous, they differ in cell size, viability, and cytoplasmic content. Viable cells are found mostly in the periphery of the sphere, while apoptosis mainly occurs in the inner part of the sphere, indicating hypoxia. When studying the neurospheres in high magnification, cilia-like cytoplasmic processes extended from the sphere can be visualized, as found *in vivo* in ependymal stem cells lining the ventricular system [65]. In the present study, cryosectioned Htx-eosin-stained HNPC spheres were carefully analysed regarding detailed morphology of the neurospheres. Actin filaments were stained using an actin specific Phalloidin probe in order to investigate alterations in the cytoskeleton network. SEM analysis was used to study the morphology of the surface of the spheres.

**Figure 5:**
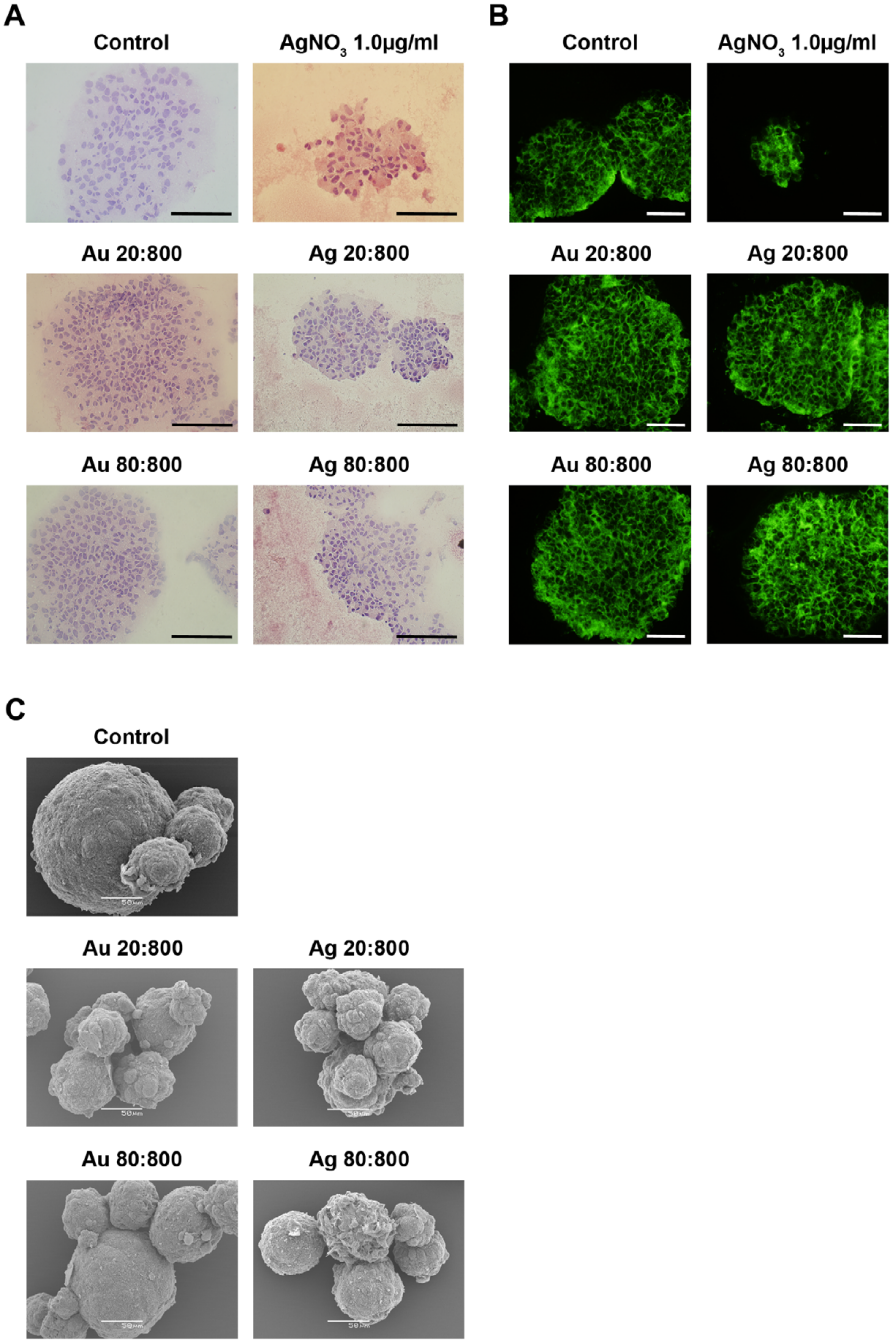
Gross morphology of HNPCs after Au- or AgNP exposure. **A.** The neurospheres were stained with Htx-eosin after NP-exposure. The NPs affected the single cell morphology seen as changed orientation of the nuclei and cytoplasm. Spheres that were exposed to NPs had increased number of nucleus and they were more centred, while the cytoplasm was turned towards the sphere edge. The NP-exposure resulted the spheres more loosen, increased the number of pyknotic cells, and induced channel formation. Au 20∶800, gold 20 nm and 800 particles/cell; Au 80∶800, gold 80 nm and 800 particles/cell; Ag 20∶800, silver 20 nm and 800 particles/cell; Ag 80∶800, silver 80 nm and 800 particles/cell; AgNO_3_ 1.0, silver nitrate 1.0 mg/ml. Scale bar equals 214 µm. **B.** The neurospheres were stained with Phalloidin after NP-exposure. NP exposure resulted in over-all altered cytoskeleton architecture, indicated by the increase in actin network. Au 20∶800, gold 20 nm and 800 particles/cell; Au 80∶800, gold 80 nm and 800 particles/cell; Ag 20∶800, silver 20 nm and 800 particles/cell; Ag 80∶800, silver 80 nm and 800 particles/cell; AgNO_3_ 1.0, silver nitrate 1.0 mg/ml. Scale bar equals 50 µm. **C.** Scanning electron microscopic evaluation of the effects of AuNPs and AgNPs on HNPCs. NPs were added to the medium 48 h after seeding and spheres were prepared for scanning electron microscopy two weeks later. NP-exposure resulted in loose aggregates and fuzzy spheres compared to control spheres, which were smooth and compact. The morphological changes were more profound with increased concentration of the NPs. However, AgNPs affected the morphology more than AuNPs. Au 20∶800, gold 20 nm and 800 particles/cell; Au 80∶800, gold 80 nm and 800 particles/cell; Ag 20∶800, silver 20 nm and 800 particles/cell; Ag 80∶800, silver 80 nm and 800 particles/cell. Scale bars equal 50 µm.

Overall, after two weeks exposure, the Au- or AgNPs affected the single cell morphology seen as changed orientation of the nuclei and cytoplasm, with the nuclei being positioned more central while the majority of the cytoplasmatic volume was turned towards the sphere edge (Fig. 5A). This phenomenon was more profound with an increased concentration of all four NPs used. AgNPs seemed to affect the sphere morphology more than AuNPs (Fig. 5A). In general, NP exposure increased the number of pyknotic cells in the spheres compared to control (Fig. 5A). However, no attempt was made to quantify numbers of nuclei or pyknotic cells. An overall decrease in density and stability of the spheres was detected after two weeks of NP exposure. Both Au- and AgNPs induced channel formation in the spheres, although most prominent after AgNP-exposure (Fig. 5A). AgNO_3_ was confirmed to be toxic as shown by the high number of pyknotic cells found in the spheres, but also as more loosely arranged spheres with fuzzy edges and extended channel formation (Fig. 5A).

Exposure to Au- or AgNPs for two weeks altered the cytoskeleton architecture and resulted in overall up-regulation of actin, shown by a more intense staining, especially at the sphere edges, when compared to control (Fig. 5B). The increase in actin-expression was more profound for spheres exposed to Ag 80∶800. Only few cells and no spheres were remaining after exposure to AgNO_3_ 5.0 µg/ml, hence no image is presented.

SEM analysis of HNPC spheres revealed a distinct change in their morphological feature after two weeks of Au- or AgNP exposure compared to control. At two weeks of NP exposure, the human spheres displayed loose and fuzzy spheres, while control spheres are smooth and compact (Fig. 5C). As mentioned above, NP exposure increased sphere aggregate formation. Only few cells and spheres were remaining after exposure to AgNO_3_ 1.0 or 5.0 µg/ml, hence no image is presented. The morphological changes were more profound with increased concentration of the NPs. However, a material effect was also evident since AgNPs affected the cellular morphology more than AuNPs.

Taken together, the detailed morphological analysis of both the gross sphere profile as well as on a cellular level, clearly demonstrate that both Au- and AgNP trigger a change in the cytoarchitecture, *i.e.* cell-to-cell contact, of the whole sphere as well as on the single cell level judged by the changed nucleus- and cytoplasm orientation.

The morphological findings from the Htx-eosin staining, actin staining, and SEM-analysis, in addition to the increased aggregation of the spheres after NP exposure, indicate both the intra cellular and surface properties may have been altered in the HNPCs. The actin fibrils are less stretched and has a more “dot”-like feature after two weeks of NP exposure, compared to control. On the contrary to our findings, others have demonstrated a decrease in actin-expression after 6 days of incubation with Au/citrate-NPs shown by immuno-labelling [66]. Soenen *et al* also showed a clear loss of actin network after AuNP exposure, but in C17.2 neural progenitor cell line the actin-loss was less prominent compared to epithelial cells [17]. Further studies may elucidate the changed expression of cell surface markers, which is highly achievable with regard to the access to well-described surface molecules. For example, integrins are important factors for maintaining functional such as cell proliferation, migration, differentiation and survival [67]. Integrins are heterodimers that consist of two transmembrane chains, one α and one β, where the β1-subunit is *e.g.* involved in the regulation in epidermal stem cell maintenance [68], [69], [70], but also neural precursor cell populations contain cells that express β1 integrins [71]. Neurons that express high levels of β1-integrin are more likely to form neurospheres than those expressing intermediate levels, although no difference in cell viability was found. Cells that express high levels of β1-integrin are found at the neurospheres edges [72]. If the neurospheres express high levels of β1-integrin, single neurospheres might stick to each other and induce aggregate formation. In further studies we will explore whether the integrin expression is changed after NP exposure of the HNPCs.

### Markers for Cellular Differentiation

**Figure 6:**
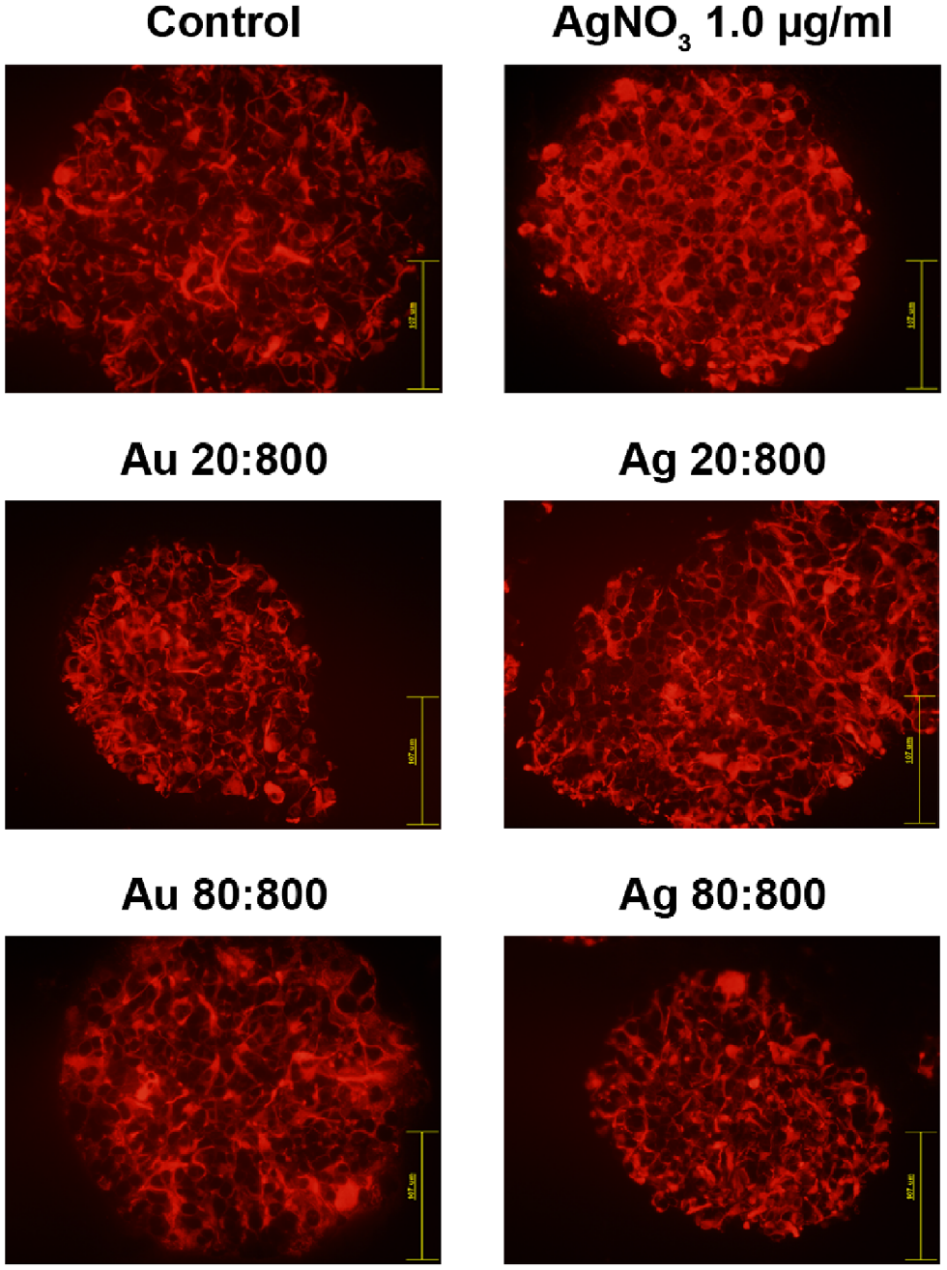
Both Au- and AgNP exposure increased the GFAP-expression in HNPCs. Exposure to the NPs resulted in longer, more perforated, and partly thicker GFAP+ processes, while nanoparticle-size or particles/cell did not have any effect the GFAP-expression. Au 20∶800, gold 20 nm and 800 particles/cell; Au 80∶800, gold 80 nm and 800 particles/cell; Ag 20∶800, silver 20 nm and 800 particles/cell; Ag 80∶800, silver 80 nm and 800 particles/cell; AgNO_3_ 1.0, silver nitrate 1.0 mg/ml. Scale bars equal 107 µm.

Since we observed a clear change in the cellular morphologies in the experimental groups, we were interested in investigating a possible accompanied alteration in the expression pattern of typically expressed early neural markers in the HNPC cultures. Immuno-labelling using GFAP- and DCX-antibodies, respectively, revealed the composition of neural cells in the neurospheres. Glial/immature neural cells express GFAP [73], [74] and neuronal progenitor cells express DCX [41], [75], [76], [77]. Exposure to Au- or AgNPs during two weeks resulted in overall up-regulation of GFAP, especially at the sphere edges, and larger cell bodies were observed compared to control (Fig. 6). A clear difference between size and concentration of the NPs was not revealed. The NP exposure also resulted in longer GFAP-positive processes. Some of the processes were thicker than the processes found in control spheres. Small spheres exposed to NPs resembled control spheres. When spheres are in a proliferative state, GFAP is found in the sphere core resembling the superficial regions of the cortex [46], [72], and upon differentiation the GFAP is homologous distributed throughout the sphere indicating a maturing cell culture. Mercury exposure to neural progenitor cells decreased the migration and increased the glial/neuron ratio [46]. In agreement, our results on increased GFAP+ staining and changed morphologies of the GFAP+ cell profiles, together with the reports by others may indicate that the NP-exposed cultures have been stimulated/stressed to begin differentiation into a more mature neural progenitor type, and most probably of glial fate.

In contrast, staining with DCX showed low levels of DCX-expression and did not reveal any obvious difference between control and NP-exposed spheres after two weeks (data not shown). Thus, the increased overall GFAP-expression, changed GFAP+ processes and no difference in DCX expression after NP exposure may indicate that Au- and AgNP induce early glial differentiation. In agreement, stressful conditions such as hypoxia induce immature neural cells to differentiate and up-regulation of GFAP expression [78], and oxidative stress caused by ozone exposure resulted in increased GFAP expression and decreased levels of DCX [79]. Minor changes, that may be detrimental in the end, in the spatial-temporal development of a neural system, can be investigated more in detail using other more refined methods that can present subtle alterations in markers for phenotypic development, such as western blotting, genomic- and proteomic techniques.

Encouragingly, the present used HNPC line has been extensively characterized with regard to it´s *in vitro* differentiation properties and is known to consistently generate a multipotential progeny. Studies are on-going using same parameters of NP exposure also on these HNPCs in differentiation conditions.

### Concluding Remarks

With the fast progress in nanotechnology, nanomedicine is also developing rapidly, but the required investigations of an eventual toxicological side-effect are yet a few steps behind. Here, we for the first time efficiently used a HNPC-based assay for investigating whether Au- or AgNPs can be taken up into the intracellular space, and whether they have a cytotoxic effect on the growth characteristics. Human neural cells can, indeed, take up both 20 and 80 nm Au- or AgNPs, respectively, exposed to the cells in very low concentrations. We demonstrate that especially AgNPs exposure cause a significant stress response in the growing HNPCs, by simultaneously affecting cell proliferation and apoptotic cell death. The composition of neural precursors in the cell line was also affected, judged by the increased expression of GFAP and the low levels of DCX expression. AuNPs, on the other hand, did not significantly affect cell proliferation or cell death, but influenced sphere morphology. Together, our results suggest that exposure to even very low concentrations of NPs may have a damaging effect on the developing human neural system and support further in-depth studies on the effect of NPs both on the developing and differentiating CNS.

## Materials and Methods

### Generation and Expansion of the Human Neural Precursor Cell Line

The human neural precursor cell line (HNPC) used for this study was originally established by L. Wahlberg, Å. Seiger, and colleagues at the Karolinska University Hospital, Stockholm, Sweden (original work with the cell line is described in [40]) and the cell line was kindly provided to us via Prof. A. Björklund (Dept. Exp. Med. Sci., Lund University, Sweden). In brief, the cell line was established from forebrain tissue, isolated and obtained from one 7-week (post conception) human embryo. Cells were cultured as free-floating neurospheres in defined DMEM-F12 medium (Invitrogen, Paisley, UK) supplemented with 2.0 mM _L-_glutamine (Sigma, St. Lois, MI, USA), 0.6% glucose (Sigma), N2-supplement (Invitrogen), 2.0 µg/ml heparin (Sigma) at 37°C in a humidified atmosphere of 5% CO_2_. Every third day, human basic fibroblast growth factor (hbFGF, 20 ng/ml; Invitrogen), human epidermal growth factor (hEGF, 20 ng/ml; PROSPEC, Rehovot, Israel), and human leukemia inhibitory factor (hLIF, 10 ng/ml; PROSPEC) were added to the culture. By using mechanical dissociation, the neurospheres were sub-cultured every 10–14 days and reseeded as single cells at a density of 1×10^5^ cell/ml. Viable cells (opalescent cells excluding Trypan Blue; Sigma) were counted in a haemocytometer. To prevent cell attachment, the flasks were gently knocked every other day. Cells passaged 10–15 times were used.

### Nanoparticles and Silver Nitrate

Prefabricated colloidal gold and silver nanoparticles (AuNPs and AgNPs) of 20 and 80 nm in diameter, respectively, dissolved in water were purchased from BBInternational (Cardiff, UK). Further dilutions were made in complete growth medium. Silver nitrate (AgNO_3_) (VWR International, Radnor, PA, USA) was dissolved in deionized water to give a stock solution of 1 mg/ml. The stock solution was sterile-filtered and stored at 4°C. Further dilutions were made in complete growth medium.

### Experimental Design

Human cells were seeded as described above, and after 48 hours AuNPs, AgNPs, or AgNO_3_ were added to the medium. Au- or AgNPs, respectively, of either 20 nm or 80 nm were added to the medium to give final concentrations of 50 or 800 particles/cell (p/c). This corresponds to final concentrations of 20 nm AuNP of 0.0004 µg/ml or 0.065 µg/ml; 80 nm AuNP of 0.026 µg/ml or 0.4 µg/ml; of 20 nm AgNP of 0.00022 µg/ml or 0.0035 µg/ml; or 80 nm AgNP of 0.014 µg/ml or 0.22 µg/ml. For imaging with electron microscopy the concentrations of 50, 800 or 2000 p/c (20 nm Au 0.016 µg/ml, 80 nm Au 1.03 µg/ml, 20 nm Ag 0.0088 µg/ml, or 80 nm Ag 0.56 µg/ml) were used for both Au- and AgNPs. AgNO_3_ was added to the medium to give final concentrations of 0.5, 1.0, and 5.0 µg/ml. The experiments were repeated three times.

After two weeks of incubation, neurospheres were either fixed or directly frozen at −20°C for morphological and histological evaluations. Prior to freezing, neurospheres were embedded for cryo-sectioning and sections of 16 µm were cut. After serial section on a cryostat, selected sections were stained for Hematoxylin-eosin (Htx-eosin). All other sections were fixated in 4% para-formaldehyde for 10 minutes at room temperature before immunocytochemistry and TUNEL staining.

### Immunocytochemistry

Cryo-sections were incubated with phosphate buffered saline containing 0.25% Triton X-100 (PBST), 1% bovine serum albumin (BSA), and 5% normal donkey serum (Jackson ImmunoResearch Laboratories Inc., West Grove, PA, USA) for 45 minutes at room temperature. Sections were incubated with primary antibody, rabbit anti-glial fibrillary acidic protein (GFAP, 1∶1500 DAKOCytomation, Glostrup Denmark), goat anti-doublecortin (DCX 1∶200, Santa Cruz Biotechnology Inc., Santa Cruz, CA, USA), and Ki67 (1∶100; Millipore, Temecula, CA, USA) overnight at 4°C and thereafter detected using Texas Red-conjugated donkey anti-rabbit IgG antibody (1∶200; Abcam, Cambridge, UK) and FITC-conjugated donkey anti-goat IgG antibody (1∶200; Jackson ImmunoResearch, West Grove, PA, USA). Sections were incubated with green fluorescent Alexa Fluor 488-conjugated Phalloidin probe (1∶200; Life Technology Europe, Stockholm, Sweden) for 2 hours at room temperature. Both primary and secondary antibodies were diluted in PBST containing 1% BSA. For counterstaining of nuclei, the sections were cover-slipped using 4′6-diamidino-2-phenylindole (DAPI)-containing Vectashield mounting medium (Vector Laboratories, Burlingame, CA, USA).

### TUNEL Assay

Cryo-sections were stained with a fluorescein-conjugated terminal deoxynucleotidyl transferase-mediated dUTP nick end-labelling (TUNEL) assay according to the manufacturer (Roche, Mannheim, Germany). For counterstaining of nuclei, the sections were cover-slipped using DAPI-containing mounting medium.

### Microscopy – Scanning Electron Microscopy (SEM) and Transmission Electron Microscopy (TEM)

For SEM, the neurospheres were fixed in 2.5% glutaraldehyde in 0.15 M Na-cacodylate buffer (pH 7.2) for 1 hour at 20°C. After rinsing in Na-cacodylate buffer, neurospheres were dehydrated in a series of ethanol with increasing concentrations, *i.e.* 50%, 70%, 96%, and 100%, prior to critical point drying (CPD030, Leica Microsystems GmbH, Kista, Sweden). Gold-Palladium (∼15 nm) was used to sputter-coat (Polaron SC7640, VG Microtech, East Sussex, UK) the preparations before imaging in a JEOL JSM-5600 LV microscope (JEOL, Japan).

For TEM, the neurospheres were fixed in 2.5% glutaraldehyde in 0.15 M Na-cacodylate buffer (pH 7.2) for 4 hour at 4°C. After rinsing in 0.1 M Na-cacodylate buffer and dehydration, the neurospheres were post-fixed in 1% osmiumtetraoxide in 0.1 M Na-cacodylate buffer at 4°C for 1 hour. After dehydration, the samples were embedded in Epon. By using an ultra microtome (Leica ultracut, Leica Microsystems GmbH, Germany) ultrathin sections were cut. The sections were stained with 2% uranyl acetate in Pb-citrate [80]. Sections were imaged in a JEOL JEM 1230 microscope (JEOL, Japan).

### Data Analysis

The growth rate after 14 days of NP exposure was estimated by counting the total numbers of viable and dead cells, respectively, after mechanical dissociation of the neurospheres. Viable cells, excluded by Trypan blue, and dead cells from three independent experiments with n = 2−3 per experimental group, were counted by using a haemocytometer.

Gross- as well as detailed morphological analysis of the neurospheres was performed using light microscopy (Nikon, Tokyo, Japan). Neurospheres, from three independent experiments, of each treatment were measured and compared to control to detect differences in size, minimum counting of 8 spheres per experimental session. Both prior to fixation and freezing, images of the neurospheres were captured for evaluation of the morphology. Counter-stained and immunostained sections of the neurospheres were examined using light- and epifluorescence microscope (Nikon Eclipse E800) equipped with appropriate filters. Images were captured with digital acquisition system (DCP Controller). Quantifications of numbers of cells expressing the proliferation marker Ki67 and the marker for apoptotic cells TUNEL, respectively, were made on 6 spheres/section and experimental group from two independent experiments. The sphere area was measured and results given as cells/mm^2^±SD. Analysis of changes of GFAP and DCX expression, respectively, were made on 6 sphere/section and experimental group from two independent experiments. Quantifications were preformed using Image J64. For TEM analysis, samples from two independent experiments were evaluated, and detailed analysis of at least 50 cells was performed. SEM analysis was made on one sample/experimental group from one culture session and approximately 20–30 spheres/sample were evaluated in the respective treatment groups.

For statistical evaluation, a two-tailed *t*-test was used. All data, when n≥3, is presented as mean±SD, and *p* values less than 0.05 were considered statistically significant.
