# Correction: Gold- and Silver Nanoparticles Affect the Growth Characteristics of Human Embryonic Neural Precursor Cells

**DOI:** 10.1371/annotation/9ecbe311-b637-4bdf-826d-48b4dd778bfa

**Published:** 2013-08-14

**Authors:** Erika Söderstjerna, Fredrik Johansson, Birgitta Klefbohm, Ulrica Englund Johansson

The Funding statement was incorrect. The correct version is:

This work was supported by grants from the Crafoord Foundation, VELUX stiftung, Clas Groschinskys Foundation, Kronprinsessan Margaretas Arbetsnämnd för synskadade (KMA), Karin Sandqvist Foundation, Maggie Stephens Foundation, Magnus Bergvall Foundation, Per Westling Foundation, and the Nanometer Consortium at Lund University. The funders had no role in study design, data collection and analysis, decision to publish, or preparation of the manuscript. 

